# The relationship between perceived teacher support and student engagement in Chinese junior high school music classrooms: the role of learning motivation

**DOI:** 10.3389/fpsyg.2026.1872072

**Published:** 2026-07-16

**Authors:** Shihan Wang, Lifang Tang

**Affiliations:** Faculty of Education, Northeast Normal University, Changchun, China

**Keywords:** junior high school students, mixed-methods research, music education, music learning motivation, perceived teacher support, student engagement

## Abstract

Student engagement in music classrooms is important because it reflects students' active participation in listening, performing, and creative activities and is associated with academic, social, and psychological outcomes. Although teacher support has been widely linked to student engagement, the indirect association between perceived teacher support, music learning motivation, and engagement in music education remains underexplored, especially in general school settings. This mixed-methods study examined associations among students' perceived teacher support, music learning motivation, and engagement in junior high school music classrooms. Survey data were collected from 568 junior high school students in urban and rural areas of Liaoning Province, Northeast China. Quantitative analyses examined associations among perceived teacher support, music learning motivation, and student engagement, and the SPSS PROCESS macro with 5,000 bootstrap samples was used to estimate the statistical indirect association. Semi-structured interviews with 15 students were analyzed through a three-level coding procedure to explain students' learning experiences. The results showed significant positive associations among perceived teacher support, music learning motivation, and student engagement. Music learning motivation was statistically associated with the link between perceived teacher support and student engagement, and the bootstrap confidence interval for the indirect association did not include zero. Qualitative findings indicated that encouragement, emotional care, clear explanation, interactive support, and content aligned with students' interests and abilities helped explain how students translated supportive classroom experiences into confidence, interest, and willingness to participate. Because the study used cross-sectional self-report data, the findings should be interpreted as associational rather than causal.

## 1 Introduction

Student engagement is widely recognized as a key determinant of students' academic achievement and psychological wellbeing, and has been shown to be significantly shaped by instructional practices (Fredricks et al., 2016). In music education, however, engagement carries particular importance because engagement in music classrooms is not only a general form of academic involvement, but also a discipline-specific form of musical practice and expression. Compared with more general forms of academic engagement, which are often reflected in students' attention to learning tasks, classroom participation, and persistence in academic work, engagement in music classrooms is more directly expressed through listening, singing, performing, creating, and emotional expression. Music learning therefore requires students not only to acquire conceptual understanding and technical skills, but also to sustain continuous participation in these musical and expressive activities. Such learning processes involve sustained attention, emotional investment, and behavioral persistence in response to performance demands and artistic expression (Schatt, 2024; Harada and Takeishi, 2025).

Furthermore, music classrooms are characterized by creative, experiential, and performative learning environments, which require students to engage at deeper cognitive, emotional, and behavioral levels compared with more knowledge-oriented disciplines (Barlow, 2018; Wang, 2021). These characteristics imply that student engagement in music education is not merely reflected in classroom participation, but also manifested through expressive performance, emotional involvement, and sustained practice. Consequently, understanding the factors that facilitate such multidimensional engagement is essential for explaining learning processes in music classrooms.

The concept of student engagement originates from early discussions on student involvement and has developed into a central construct in educational research since the mid-1990s. A substantial body of research has demonstrated that students' participation in educational activities is closely associated with a wide range of positive developmental outcomes, including academic achievement, persistence, satisfaction, and social integration (Astin, 1984; Trowler, 2010).

Student engagement is generally conceptualized as a multidimensional construct comprising behavioral, emotional, and cognitive dimensions (Fredricks et al., 2004). Behavioral engagement refers to students' observable participation, effort, attention, and persistence; emotional engagement reflects students' affective reactions, sense of belonging, and identification with the learning context; and cognitive engagement involves the use of deep learning strategies and self-regulated learning processes (Finn, 1989; Voelkl, 1997; Pintrich and de Groot, 1990; Zimmerman, 1990).

In classroom-based research, engagement is typically understood as students' active involvement in specific instructional settings rather than their general participation in school life (Cents-Boonstra et al., 2021). In music education, student engagement specifically refers to students' active participation in listening, singing, performing, and creative activities, as well as their sustained involvement in music learning processes (Barlow, 2018). Additionally, some researchers conceptualize engagement as a positive and persistent psychological state characterized by vigor, dedication, and absorption, reflecting a more dynamic understanding of students' learning experience (Schaufeli et al., 2002; Wei et al., 2025).

Among the various factors influencing student engagement, perceived teacher support has been consistently identified as a crucial contextual variable. Perceived teacher support refers to students' subjective perceptions of the assistance, guidance, and emotional and instructional resources provided by teachers to facilitate learning (Martin et al., 2024). Within instructional contexts, teacher support extends beyond academic scaffolding to include the creation of a supportive classroom climate, autonomy-enhancing practices, and individualized responsiveness to student needs (Held and Mori, 2024). From a self-determination theory perspective, such support plays a role in satisfying students' basic psychological needs for autonomy, competence, and relatedness, thereby shaping their motivational and behavioral engagement in learning activities.

Early research conceptualized teacher support in terms of involvement, structure, and autonomy support (Skinner and Belmont, 1993), which correspond to teachers' roles in fostering relationships, organizing classroom environments, and promoting student autonomy. Subsequent studies have further highlighted the multidimensional nature of teacher support, including emotional support, instrumental support, autonomy support, and structural support within the framework of self-determination theory (Strati et al., 2017; Zhou et al., 2022).

A substantial body of empirical research has consistently demonstrated a positive association between perceived teacher support and student engagement. Supportive teaching practices and high-quality teacher–student relationships have been associated with higher levels of students' behavioral participation, emotional involvement, and cognitive engagement in classroom learning (Klem and Connell, 2004; Cents-Boonstra et al., 2021). Longitudinal evidence further indicates that emotional and instructional support from teachers can be positively associated with students' subsequent engagement over time (Hughes et al., 2008; Ruzek et al., 2016). A recent meta-analysis also confirms a robust association between teacher support and engagement across educational contexts, particularly in emotional engagement domains (Vargas-Madriz et al., 2024). Collectively, these findings suggest that teacher support constitutes a stable contextual factor closely linked to students' engagement experiences.

Research has shown that teachers' ability to build relationships, connect with students through music, and empower learners is closely associated with students' learning experiences and their willingness to continue engaging in music learning (Robinson, 2018). In more specific classroom settings, teachers' instructional arrangements, guidance strategies, and supportive behaviors have been found to be closely associated with students' participation in music classrooms (Jiang et al., 2025). Moreover, studies conducted among Chinese university music students indicate that autonomy-supportive teaching is significantly associated with students' immersion, collaborative awareness, and wellbeing, further highlighting the importance of teacher support in music education contexts (Xu and Li, 2025).

Although perceived teacher support is consistently associated with student engagement, the underlying psychological mechanisms through which this relationship operates remain insufficiently understood, particularly in the context of music education. Learning motivation refers to the underlying reasons that drive students to engage in and persist with learning activities, influencing the direction, intensity, and quality of their efforts (Maehr and Meyer, 1997; Appleton et al., 2006; Comeau et al., 2019), while student engagement reflects the observable manifestation of these motivational processes in action (Reeve et al., 2004; Frydenberg et al., 2005). Existing studies have often examined these associations in general academic or non-music subject contexts, leaving the motivational processes underlying engagement in music classrooms relatively underexplored (Tas et al., 2019; An et al., 2022; Zhang and Hu, 2025). Given the performative, expressive, and emotionally intensive nature of music learning, it remains unclear whether and how perceived teacher support is statistically associated with student engagement through motivational processes in this specific instructional context. Addressing this gap is essential for advancing both theoretical understanding and subject-specific applications of student engagement research.

Self-determination theory (SDT) provides a comprehensive framework for understanding this mechanism. According to SDT, motivation exists along a continuum ranging from amotivation to intrinsic motivation, with various forms of extrinsic motivation reflecting different degrees of internalization, including external regulation, introjected regulation, identified regulation, and integrated regulation (Ryan and Deci, 2000, 2017; Vallerand et al., 1992). The theory further posits that the satisfaction of three basic psychological needs—autonomy, competence, and relatedness—facilitates the development of more autonomous forms of motivation (Ryan and Deci, 2002; Evans, 2015).

In supportive classroom environments, teachers can foster students' psychological need satisfaction, thereby supporting autonomous motivation, which is associated with higher levels of engagement (Praetorius et al., 2018; Zhou et al., 2022). Empirical evidence is consistent with this indirect-association pattern. For example, Tas et al. (2019) found that students' perceptions of teacher support were positively associated with task value and academic self-concept, and that these motivational beliefs were in turn positively associated with multiple dimensions of engagement. Similarly, studies in technology-integrated classrooms and foreign language learning contexts have reported an indirect association between teacher support and student engagement through learning motivation (An et al., 2022; Zhang and Hu, 2025). In addition, Jang (2008) demonstrated that autonomy-supportive teaching enhances identified regulation, leading to increased behavioral engagement. These findings suggest that learning motivation may be an important psychological variable associated with the link between teacher support and student engagement.

In music education, learning motivation plays an especially critical role due to the long-term, effortful, and practice-oriented nature of music learning. Motivation is associated not only with students' learning experiences and academic performance but also with their emotional wellbeing and willingness to continue engaging in music activities (Evans and Liu, 2019). Within this field, self-determination theory has been widely recognized as a dominant framework for understanding music learning motivation (Evans, 2015).

Research has shown that more autonomous forms of motivation, particularly intrinsic motivation and identified regulation, are closely associated with higher levels of engagement, academic achievement, and positive learning experiences (Bureau et al., 2022). Moreover, instructional practices play a key role in shaping students' motivation. Systematic review studies indicate that self-determination theory and expectancy–value theory are among the most frequently used frameworks in research on music learning motivation, and teachers' instructional strategies are critical in enhancing students' motivation (Kiss et al., 2025).

Specifically, autonomy-supportive teaching practices—such as providing meaningful choices, offering clear rationales, and acknowledging students' perspectives—have been shown to foster more positive learning experiences and better learning outcomes in music classrooms (Bonneville-Roussy et al., 2020). Learner-centered teaching approaches that promote social interaction, autonomy, and meaningful participation are also effective in sustaining long-term engagement in music learning (Krause and Davidson, 2018). Furthermore, recent research indicates that when music classrooms support students' needs for autonomy, competence, and relatedness, students demonstrate higher levels of intrinsic motivation, greater classroom participation, and stronger persistence in music learning (Schatt, 2024).

Although SDT distinguishes among different types of motivation, this study focused on the overall level of music learning motivation during students' participation in music classroom learning. This treatment was consistent with the research purpose, which was to examine the association between perceived teacher support, music learning motivation, and student engagement at the overall construct level. At the same time, the reliability and validity of the revised music learning motivation scale were examined to provide further support for this treatment.

Despite the substantial body of research demonstrating the positive relationship between teacher support and student engagement, as well as the indirect association involving learning motivation in general educational and non-music subject contexts (Tas et al., 2019; An et al., 2022; Zhou et al., 2022; Zhang and Hu, 2025), empirical research focusing specifically on music classrooms remains relatively limited. In particular, few studies have examined the associations among perceived teacher support, learning motivation, and student engagement within a unified analytical framework in Chinese junior high school music classrooms. As a result, it remains unclear whether the association between perceived teacher support and student engagement is statistically linked to motivational processes in this specific instructional context.

To address this gap, the present study examines the relationships among perceived teacher support, learning motivation, and student engagement in Chinese junior high school music classrooms, and further tests whether learning motivation is statistically associated with the link between perceived teacher support and student engagement.

Specifically, this study addresses the following research questions: (1) Is perceived teacher support positively associated with student engagement in music classrooms? (2) Is perceived teacher support positively associated with music learning motivation? (3) Is music learning motivation positively associated with student engagement? (4) Is there an indirect association between perceived teacher support and student engagement through music learning motivation?

Based on self-determination theory, the following hypotheses are proposed:

H1: Perceived teacher support is positively associated with student engagement in music classrooms.
H2: Perceived teacher support is positively associated with music learning motivation.
H3: Music learning motivation is positively associated with student engagement.
H4: Perceived teacher support is indirectly associated with student engagement through music learning motivation.

## 2 Methods

### 2.1 Research design

This study adopted a mixed-methods research design, integrating quantitative and qualitative approaches to comprehensively examine the relationships among perceived teacher support, music learning motivation, and student engagement in music classrooms. Specifically, an explanatory sequential design was employed, in which quantitative data were first collected and analyzed, followed by qualitative inquiry to further interpret and explain the quantitative findings.

In the quantitative phase, multiple statistical techniques were used to test the proposed hypotheses. These included descriptive statistics, reliability analysis, confirmatory factor analysis (CFA), convergent and discriminant validity testing, common method bias testing, correlation analysis, regression analysis, and mediation analysis. These methods allowed for a systematic examination of the structural relationships among the key variables.

In the qualitative phase, semi-structured interviews were conducted to gain a deeper understanding of how students perceived teacher support and how they related such perceptions to motivation and engagement in music classroom learning. By integrating quantitative and qualitative findings, this study not only examined the hypothesized associations but also provided a more nuanced explanation of the classroom processes underlying these associations.

### 2.2 Participants

#### 2.2.1 Quantitative sample

The participants in the quantitative phase consisted of junior high school students enrolled in music classes. A combination of random sampling and stratified sampling was employed to ensure representativeness.

First, four junior high schools in Liaoning Province, Northeast China, were randomly selected. Considering the potential disparities between urban and rural education contexts in China, two schools were selected from urban areas and two from rural areas based on administrative division standards. Subsequently, stratified sampling was conducted by grade level, and students from Grade 7 and Grade 8 were randomly selected to participate in the survey.

A total of 594 questionnaires were distributed. After data screening, 26 questionnaires were excluded due to invalid response patterns (e.g., identical responses across all items or inconsistent answers to repeated questions). The final valid sample consisted of 568 questionnaires, yielding an effective response rate of 95.6%.

The sample included 279 male students (49.12%) and 289 female students (50.88%). Prior to data collection, all participants were informed of the research purpose and assured of confidentiality and anonymity. Participation was voluntary, and all questionnaires were completed in Chinese to avoid potential bias caused by translation.

#### 2.2.2 Qualitative sample

In the qualitative phase, maximum variation sampling was used to select 15 participants from students who had completed the questionnaire and expressed willingness to participate in follow-up interviews. The interview sample covered all four participating schools, with 4, 4, 3, and 4 students selected from the four schools, respectively. Eight students were from urban schools and seven were from rural schools; seven students were in Grade 7 and eight were in Grade 8; and the sample included eight boys and seven girls. This sampling strategy was intended to capture diverse perspectives across school context, grade level, and gender.

Semi-structured interviews were conducted with the selected students, with each interview lasting approximately 15 min. All interviews were conducted in Chinese and audio-recorded with participants' consent.

### 2.3 Measures

#### 2.3.1 Perceived teacher support scale

Students' perceived teacher support was measured using an adapted version of the Perceived Teacher Support Scale, which has demonstrated good reliability and validity in previous studies (Patrick et al., 2007). The original scale includes two dimensions: teacher emotional support and teacher academic support, with four items in each dimension.

To better align with the music classroom context, this study revised the wording of the original items to reflect music learning situations, resulting in the Music Classroom Perceived Teacher Support Scale. The adapted scale consisted of 8 items while retaining the original two-dimensional structure.

All items were rated on a five-point Likert scale ranging from 1 (“almost never”) to 5 (“always”). Higher scores indicated higher levels of perceived teacher support in the music classroom.

#### 2.3.2 Student engagement scale

Student engagement in music classrooms was measured using an adapted version of the Learning Engagement Scale developed by Fang et al. (2008), which is based on the Utrecht Work Engagement Scale–Student version (UWES-S) originally proposed by Schaufeli et al. (2002).

The Chinese version developed by Fang et al. (2008) has been widely used and validated in educational contexts in China. In the present study, the wording of the items was further modified to fit the context of music classroom learning, resulting in the Music Classroom Engagement Scale.

The scale retained the three dimensions of vigor, dedication, and absorption and included 17 items. All items were rated on a five-point Likert scale ranging from 1 (“strongly disagree”) to 5 (“strongly agree”). Higher scores indicated higher levels of student engagement in music classrooms.

#### 2.3.3 Motivation for learning music scale

Music learning motivation was measured using an adapted version of the Motivation for Learning Music (MLM) questionnaire developed by Comeau et al. (2019), which is grounded in self-determination theory.

To ensure contextual relevance, all items were revised to begin with the unified stem “I participate in music classroom learning.” In addition, several modifications were made to improve the construct validity of the scale.

First, two items related to parental influence (original Items 3 and 12) were removed, as this study focuses specifically on classroom-based learning motivation rather than family-related factors. Second, to reduce conceptual overlap with the perceived teacher support construct and to maintain a clearer distinction between perceived teacher support and music learning motivation in the statistical indirect-association model, two items (original Items 9 and 22) related to concerns about teachers' expectations were also excluded.

The revised MLM scale included 21 items and was used to assess students' motivation in music classroom learning. All items were rated on a five-point Likert scale ranging from 1 (“strongly disagree”) to 5 (“strongly agree”).

Although the original music learning motivation questionnaire was based on the multifaceted motivational structure of self-determination theory, this study focused on students' overall level of music learning motivation during classroom learning. In the main analysis, the adjusted scale was considered as an overall latent construct, and its reliability and validity were assessed. The test results supported this treatment. Specifically, Cronbach's α = 0.962, composite reliability (CR) = 0.962, average variance extracted (AVE) = 0.548, and the standardized factor loadings ranged from 0.707 to 0.770.

#### 2.3.4 Semi-structured interviews

Semi-structured interviews were conducted to complement the quantitative data and provide deeper insights into students' learning experiences. The interview protocol focused on four key aspects: (1) students' overall perceptions of music classroom learning, (2) perceived teacher support, (3) music learning motivation, and (4) manifestations of student engagement.

In addition, reflective questions were included to explore how students interpret teacher support and how they connect it with their motivation and engagement, as well as to collect suggestions for improving music classroom teaching.

Each interview lasted approximately 15 min and was conducted in Chinese.

### 2.4 Data collection procedure

This study adopted a mixed-methods approach and used four research tools: a perceived teacher support questionnaire, a music learning motivation questionnaire, a music classroom engagement questionnaire, and a semi-structured interview outline. All questionnaires and interview tools were presented in Chinese.

In line with the current situation of music classrooms in junior high schools, the three questionnaire scales were adjusted for the present study. The adjustment process included three distinct stages. First, the original items were sorted out and adjusted, and general learning terms were replaced with music classroom-specific terms, such as listening, singing, performing, practicing, and participating in music classroom activities, while retaining the original construct meaning. Two researchers with backgrounds in music education and educational psychology reviewed the adjusted Chinese items to evaluate the relevance of their content, the clarity of the expression, and the fit with the intended constructs. A pre-test was also conducted with 54 junior high school students, with the aim of examining whether each item was easy to understand and in line with the cognitive level of the target participants. Small changes were made to the content of the questionnaire according to the feedback from research team review and pre-testing.

The study was reviewed and approved by the Science and Technology Ethics Committee of Northeast Normal University (approval no. 202602011). Before data collection began, the researchers explained the study's purpose to the participants and clearly informed them that questionnaire completion and interview participation were voluntary. All data were used only for academic research and were kept strictly confidential to ensure the privacy and anonymity of participants. The quantitative research part was divided into two implementation stages: a pilot study and a main study. Using the data collected from the aforementioned 54 students during the pilot stage, the results showed that the standardized Cronbach's α coefficients for the three scales were 0.808, 0.850, and 0.785, respectively, and that the KMO values for the three scales were 0.798, 0.837, and 0.944, respectively. In addition, Bartlett's test of sphericity was significant (p < 0.001). These preliminary results indicated acceptable initial reliability and suitability for further analysis. After the pre-test was completed, the researchers conducted formal quantitative data collection across the four participating junior high schools. The questionnaires were distributed in paper form with the assistance of the class teachers and completed by students on site. After the quantitative data collection was completed, the returned questionnaires were screened, and valid responses were retained for subsequent statistical analysis. In the formal study, the three scales showed good internal consistency; the results of the confirmatory factor analysis indicated that the measurement model fit the data well. This supported the reliability and construct validity of the tools used.

After the quantitative study was completed, 15 students who had completed the questionnaire and were willing to participate in follow-up interviews were selected. The interview outline was designed around the purpose and core variables of this study. Necessary language adjustments were made before the formal interview. Before the formal interviews, three junior high school students participated in a small-scale pilot interview to ensure that the questions were clear and age-appropriate. Formal one-on-one interviews were then conducted with the 15 selected students. The interviews were conducted in a relaxed atmosphere to encourage students to express their views on teacher support, music learning motivation, and classroom engagement. With students' consent, the interviews were audio-recorded and transcribed verbatim for analysis.

### 2.5 Data analysis

The quantitative data in this study were analyzed using SPSS 26.0. First, descriptive statistics were conducted to examine the overall distribution and central tendencies of the key variables, including students' perceived teacher support, music learning motivation, and student engagement in music classrooms. Subsequently, reliability analysis (Cronbach's α) and confirmatory factor analysis (CFA) were performed to assess the internal consistency and structural validity of the measurement scales. Common method bias was then assessed using the Harman single-factor test and the full collinearity variance inflation factor (VIF) test.

Next, correlation analysis was conducted to explore the associations among the main variables. Based on these results, multiple regression analyses were employed to examine the associations between perceived teacher support, music learning motivation, and student engagement. Finally, mediation analysis was conducted using the SPSS PROCESS macro with 5,000 bootstrap samples to evaluate the proposed statistical indirect association.

In the qualitative phase, all interview recordings were first transcribed verbatim into textual data. The data were then analyzed using a three-level coding procedure, including open coding, axial coding, and selective coding.

During open coding, the two coders independently read and coded the transcripts, sorting and screening meaningful entries related to students' perceptions of teacher support, music learning motivation, and student engagement. In response to differences in the initial coding, the two coders fully communicated and discussed until a consensus was formed. If the coding was unclear, it was reviewed against the original interview record. During axial coding, concepts with similar characteristics were grouped into higher-level categories. At the same time, the number of students linked to each theme category was counted and retained. During selective coding, the core themes were further refined around the research topic. After the relevant content was integrated, it was used to explain how students related the support provided by teachers to self-confidence, interest, willingness to participate, and student engagement in the music classroom. Across the 15 interviews, recurring patterns were identified, and the construction logic of the coding framework and the development process of the core themes are shown in Table 1.

**Table 1:** Qualitative coding structure and theme recurrence.

Selective theme	Axial categories	Example initial codes	Students contributing
Interest and enjoyable music-class experience	Enjoyment; relaxation; contrast with examination-oriented subjects	“music class is fun”; “music makes me relaxed”; “less exam-oriented”; “I feel happy in music class”	15/15
Teacher support and encouragement	Emotional reassurance; confidence-building; instructional guidance	“teacher helped me calm my nervousness”; “teacher encouraged me to go on stage”; “I became more confident in singing”; “teacher gave targeted guidance”	14/15
Attractive classroom activities	Performance opportunities; instrumental practice; video-supported learning; interaction	“I like stage singing”; “instrumental activities are vivid and interesting”; “I am eager to participate”; “videos make class more enjoyable”	13/15
Alignment with interests and abilities	Interest relevance; accessible difficulty; content-ability fit	“content interests us”; “songs are within my ability”; “some songs are too high to sing”; “I will be very active if I can sing it”	10/15

To enhance the transparency and trustworthiness of the analysis, representative quotations from the interviews were used to support each identified theme. The interview transcripts and coding results were reviewed repeatedly. This helped ensure consistency between the extracted themes and the original data. Peer debriefing was also conducted. A researcher with relevant expertise reviewed portions of the interview data and coding process, and disagreements were discussed until a shared interpretation was reached.

## 3 Results

### 3.1 Quantitative phase

The quantitative phase used questionnaire data from 568 valid participants to examine the relationships among students' perceived teacher support, music learning motivation, and music classroom engagement. The analyses included reliability analysis, confirmatory factor analysis (CFA), common method bias testing, descriptive statistics, correlation analysis, regression analysis, and mediation analysis.

#### 3.1.1 Reliability and validity of the measurement tools

First, the measurement tool's internal consistency was assessed using Cronbach's alpha. The results showed that the Cronbach's α coefficients for students' perceived teacher support, music classroom engagement, and music learning motivation were 0.826, 0.875, and 0.962, respectively, indicating good internal consistency for each component. Since all the measurement tools used in this study were adapted from existing, mature scales with clear theoretical dimensions, a confirmatory factor analysis (CFA) was conducted to evaluate the structural validity of each indicator. The results showed that the measurement model fit the data well: χ^2^/df = 1.246, AGFI = 0.904, GFI = 0.913, IFI = 0.984, TLI = 0.983, CFI = 0.984, RMSEA = 0.021 (see Table 2). In addition, the standardized factor loadings for each indicator ranged from 0.670 to 0.888; the CR ranged from 0.866 to 0.962; and the AVE ranged from 0.518 to 0.660, indicating satisfactory convergent validity for each component (see Table 3). Furthermore, the square root of the AVE for all latent variables was greater than the correlation coefficient between each latent variable and other components, thus confirming that each component had good discriminant validity.

**Table 2:** Model fit indices for the measurement model.

Fit index	Recommended value	Observed value
χ^2^/df	1–3	1.246
AGFI	>0.80	0.904
GFI	>0.80	0.913
IFI	>0.90	0.984
TLI	>0.90	0.983
CFI	>0.90	0.984
RMSEA	< 0.08	0.021

**Table 3:** Psychometric properties of the adapted measurement scales.

Construct/dimension	Items	Cronbach's alpha	Standardized loading range	AVE	CR
Perceived teacher support	8	0.826	0.725-0.888	0.657-0.660	0.884-0.885
Teacher emotional support	4	0.883	0.725-0.873	0.657	0.884
Teacher academic support	4	0.884	0.747-0.888	0.660	0.885
Music classroom engagement	17	0.875	0.670-0.877	0.518-0.622	0.866-0.891
Vigor	6	0.868	0.708-0.745	0.524	0.869
Dedication	5	0.890	0.670-0.877	0.622	0.891
Absorption	6	0.866	0.694-0.752	0.518	0.866
Music learning motivation	21	0.962	0.707-0.770	0.548	0.962

#### 3.1.2 Common method bias test

The Harman single-factor test was used to assess the potential impact of common method bias. The results showed that the unrotated first factor explained 32.178% of the total variance, which was below the 40% threshold, suggesting that serious common method bias was not indicated by this test. In addition, regression analyses were conducted for each core variable against the other core variables. The full collinearity variance inflation factor (VIF) test was also completed. The VIF values ranged from 1.231 to 1.281, which were lower than the recommended threshold of 3.3. Taken together, these results suggest that common method bias was unlikely to have a serious effect on the findings. However, because all main quantitative variables were collected from student self-report questionnaires at the same time point, common method bias cannot be fully ruled out.

#### 3.1.3 Descriptive statistics and correlation analysis

Table 4 presents the means, standard deviations, reliability coefficients, and correlation coefficients for the main study variables. Students' perceived teacher support was positively related to music classroom engagement (*r* = 0.468, *p* < 0.01) and music learning motivation (*r* = 0.433, *p* < 0.01). In addition, music learning motivation was positively related to music classroom engagement (r = 0.448, *p* < 0.01). These results provide preliminary support for hypotheses 1 to 3.

**Table 4:** Means, standard deviations, reliabilities, and correlations among the main study variables.

Variable	M	SD	α	1	2	3
1. Students' perceived teacher support	3.037	0.581	0.826	1		
2. Music classroom engagement	3.357	0.526	0.875	0.468^**^	1	
3. Music learning motivation	3.500	0.734	0.962	0.433^**^	0.448^**^	1

^**^p < 0.01.

#### 3.1.4 Hypothesis testing

Regression analyses were conducted to test hypotheses 1 to 3. Students' perceived teacher support was positively associated with their music classroom engagement (B = 0.424, *p* < 0.001), thus supporting Hypothesis 1. In addition, students' perceived teacher support was positively associated with their music learning motivation (B = 0.547, *p* < 0.001), thus supporting Hypothesis 2. The detailed regression results are presented in Table 5.

**Table 5:** Regression analysis results.

Predictor	Music classroom engagement (Model 1) B	*t*	*p*	Music learning motivation (Model 2) B	*t*	*p*	Music classroom engagement (Model 3) B	*t*	*p*
Constant	2.070	19.922	< 0.001	1.840	12.437	< 0.001	1.672	14.978	< 0.001
Students' perceived teacher support	0.424^***^	12.612	< 0.001	0.547^***^	11.429	< 0.001	0.306^***^	8.608	< 0.001
Music learning motivation	—	—	—	—	—	—	0.216^***^	7.688	< 0.001
R^2^	0.219			0.188			0.293		
Adjusted R^2^	0.218			0.186			0.291		
F	159.056		< 0.001	130.623		< 0.001	117.241		< 0.001

B = unstandardized coefficient. ^***^p < 0.001.

When students' perceived teacher support and music learning motivation were included in the regression model with music classroom engagement as the outcome, both perceived teacher support (B = 0.306, *p* < 0.001) and music learning motivation (B = 0.216, *p* < 0.001) were positively associated with engagement. Therefore, Hypothesis 3 was supported.

#### 3.1.5 Mediation analysis

This study analyzed the statistical indirect association using Model 4 of the SPSS PROCESS macro with 5,000 bootstrap samples to examine whether music learning motivation was statistically associated with the relationship between students' perceived teacher support and music classroom engagement. The reported effects were unstandardized coefficients. The mediation analysis results are presented in Table 6. The results showed that the indirect association was 0.118, with a 95% confidence interval of [0.084, 0.151], excluding zero, indicating a significant indirect association. At the same time, the direct association between students' perceived teacher support and their music classroom engagement remained significant (effect = 0.306, 95% confidence interval [0.236, 0.375], p < 0.001), and the total association was 0.424 (95% confidence interval [0.358, 0.490], p < 0.001). These results indicate that a partial indirect association was observed between students' perceived teacher support and their music classroom engagement through music learning motivation, thus supporting Hypothesis 4. Given the cross-sectional design, the mediation model should be interpreted as a statistical indirect association rather than a causal pathway. To further examine the sensitivity of the results, two alternative mediation models were also analyzed. In the first model, music classroom engagement was tested as the mediator between perceived teacher support and music learning motivation. In the second model, perceived teacher support was tested as the mediator between music learning motivation and music classroom engagement. Both alternative models showed statistically significant indirect associations. The indirect association was 0.1857, 95% CI [0.1365, 0.2410], in the first alternative model and 0.1048, 95% CI [0.0769, 0.1355], in the second alternative model. Compared with the proposed model, in which the indirect association was 0.118, the first alternative model showed a larger indirect coefficient, whereas the second alternative model showed a smaller but still statistically significant indirect coefficient. These results suggest that several directional arrangements were statistically plausible in the cross-sectional data; therefore, the proposed model should be interpreted as a theoretically guided statistical indirect-association model rather than evidence of a single causal pathway.

**Table 6:** Mediation analysis of music learning motivation.

Path	Effect type	Effect	95% CI Lower	95% CI Upper	*SE*	*p*
Students' perceived teacher support → Music learning motivation → Music classroom engagement	Indirect effect	0.118	0.084	0.151	0.017	—
Students' perceived teacher support → Music classroom engagement	Direct effect	0.306	0.236	0.375	0.036	< 0.001
Students' perceived teacher support → Music classroom engagement	Total effect	0.424	0.358	0.490	0.034	< 0.001

Effects are reported as unstandardized coefficients. CI, confidence interval.

In summary, the quantitative results showed positive associations among students' perceived teacher support, music learning motivation, and music classroom engagement. Music learning motivation was statistically associated with the link between perceived teacher support and engagement, but the directionality of these associations cannot be established from the present cross-sectional data.

### 3.2 Qualitative phase

Analysis of interview data from 15 students identified four interrelated themes: (1) students' intrinsic interest and the enjoyable nature of music classes, (2) teachers' support and encouragement, (3) the attractiveness of classroom activities, and (4) the alignment between teaching content and students' interests and abilities. During the coding process, 15 students expressed feelings related to interest or enjoyment, 14 students discussed teachers' encouragement or emotional/teaching support, 13 students mentioned interactive or experiential classroom activities, and 10 students clearly associated their degree of participation with teaching content aligned with their interests or ability levels. Taken together, these findings clarify how students experienced the statistical associations observed in the quantitative phase. Table 1 summarizes the qualitative coding structure and shows how students' interview narratives were developed from initial codes into core categories and final themes.

#### 3.2.1 Interest and enjoyable experience of music classes

First, students' engagement in music classrooms was strongly grounded in their interest in the subject and the relaxed, enjoyable learning atmosphere. Many students emphasized that music classes differ from other academic subjects in that they are less rigid and more engaging. For example, S2 noted: “I like it very much. I think music is a lot of fun. It makes me happy physically and mentally.” S2 further explained that music class is “not only about learning, but also a kind of physical and mental relaxation.” Similarly, S1 described music classes as “relatively relaxed… less rigid than academic subjects… overall, I feel quite happy.” S10 characterized music class as “less exam-oriented and more relaxing,” highlighting its role in alleviating the pressure of other courses. S11 also reported that music classes are “very relaxed and pleasant,” contributing to a generally positive learning experience.

These responses suggest that the sense of enjoyment, relaxation, and aesthetic experience inherent in music classes constitutes a fundamental condition for sustained student engagement. These quotations mainly reflect students' music learning motivation, especially their interest and enjoyment in music classroom learning, and provide a motivational background for understanding their sustained engagement.

#### 3.2.2 Teacher support and encouragement

Second, teacher support and encouragement emerged as important factors related to students' engagement. Participants repeatedly emphasized that teachers' encouragement, patience, emotional care, and respectful communication were related to their confidence and willingness to participate. For instance, S2 explained: “When I lack confidence in singing, the teacher first helps calm my nervousness and then gives targeted guidance.” When asked how this support was related to his learning experience, S2 added: “It means a lot to me… I would feel more confident and learn better.” Similarly, S3 shared: “When I feel socially anxious, the teacher encourages me to go on stage and reminds me that everyone feels nervous, so I should just stay calm.” S7 also noted that the teacher reassured her about her pitch ability, which helped her “gradually open up and perform more confidently.”

In terms of emotional support, S1 mentioned that when feeling upset, the teacher would “check on me privately after class, which makes me feel very warm and supported.” Likewise, S2 described the teacher as “very attentive and considerate,” noting that the teacher could quickly perceive emotional changes and offer timely support.

These quotations directly illustrate perceived teacher support, including emotional reassurance, encouragement, and instructional guidance. They also show how such support was related to students' confidence, emotional security, and willingness to participate, thereby connecting perceived teacher support with music learning motivation and student engagement.

#### 3.2.3 Attractiveness of classroom activities

Third, the attractiveness and participatory nature of classroom activities were closely related to students' willingness to engage. Students expressed a strong preference for interactive and experiential activities, such as stage performances, group singing, gesture-based movement, instrumental practice, music listening, and video-based learning.

For example, S2 stated: “I really like the stage singing activities… they allow me to express myself confidently.” S3 similarly reported enjoying “the feeling of performing on stage” and highlighted instrumental activities as “vivid and interesting,” enabling a direct experience of musical enjoyment. S5 noted that instrumental practice sessions, supported by teacher demonstrations and video guidance, created a “relaxed and enjoyable classroom atmosphere.” S8 added: “As long as there is an activity, I am eager to participate and express myself.”

At the same time, individual preferences varied. S4 preferred “teachers explaining while playing music” and emphasized learning through listening, while S6 expressed a preference for video-based instruction. S9 highlighted that gesture-based activities created a “lively classroom atmosphere” that enhanced attention and engagement.

These quotations mainly connect attractive classroom activities with music learning motivation and student engagement. Students' enjoyment of stage singing, instrumental practice, video-supported learning, and classroom interaction reflected their interest in music learning, while their eagerness to participate and express themselves reflected behavioral and emotional engagement. In addition, S5′s account of teacher demonstrations and video guidance also links classroom activities with instructional support.

#### 3.2.4 Alignment between teaching content and students' interests and abilities

Finally, students' engagement was closely related to the degree to which teaching content aligned with their interests, abilities, and learning needs. Students were more likely to engage actively when the content was accessible, relevant, and personally meaningful.

S1 noted: “As long as the teacher is enthusiastic and the content interests us, I will be very engaged and willing to participate.” This quotation links teacher enthusiasm and content interest with the student's willingness to participate, thereby connecting perceived teacher support, music learning motivation, and student engagement in one classroom account. S4 expressed a preference for teaching approaches that integrate music listening into instruction, while S10 favored demonstration-based teaching, stating that repeated modeling by the teacher supported learning.

Conversely, when the content exceeded students' ability levels, engagement tended to decline. S7 reported that some songs were “too high to sing,” which reduced her interest, while S5 noted that “if the songs are within my ability, I will be very active.”

These quotations show that content aligned with students' interests and abilities was related to music learning motivation and classroom engagement. When songs were within students' ability levels, students described themselves as more active; when songs exceeded their ability levels, students reported reduced interest.

#### 3.2.5 Summary of qualitative findings

Overall, student engagement in music classrooms was associated with the combined presence of multiple factors, including intrinsic interest, teacher support, classroom activities, and the alignment between content and learner characteristics. Among these, teacher support played a particularly central role, as students connected supportive teacher behaviors with classroom experiences, interest, confidence, and willingness to participate.

For example, S2 reported that after receiving encouragement, “I became more confident in singing, so I could learn better,” while S8 noted that liking the teacher was related to their willingness to participate. S10 further observed that teachers' emotional states were closely related to classroom dynamics, indicating that students are highly sensitive to teachers' attitudes and behaviors. These examples clarify how different quotations correspond to the study's core variables: S2 illustrates perceived teacher support and confidence-building, S8 links teacher-related preference with willingness to participate, and S10 shows students' sensitivity to teachers' emotional and behavioral support in classroom interaction.

In summary, these findings show that teacher support was not experienced by students in isolation. Rather, students' accounts linked teachers' supportive behaviors with greater self-confidence, interest, and willingness to participate, thereby providing a qualitative explanation for the statistical indirect association among perceived teacher support, music learning motivation, and student engagement in music classrooms.

### 3.3 Data Integration

Data integration was an important feature of mixed-methods research (Nassaji, 2025). In this study, data integration was further reflected in the connection between the quantitative model and the qualitative themes. Quantitative analysis showed that students' perceived teacher support was positively correlated with music learning motivation and student engagement in music classrooms. It also showed a significant statistical association among teacher support, learning motivation, and engagement. Qualitative analysis, through students' descriptions, clarified how teachers' encouragement, emotional care, clear explanations, and opportunities for participation were related to students' self-confidence, interest, and willingness to participate, thereby helping to explain this model.

Table 7 shows the joint display used to integrate quantitative model components and qualitative themes. This table does not simply repeat the quantitative results. Instead, it shows how the interview findings help interpret the statistical model.

**Table 7:** Mixed-methods integration of quantitative results and qualitative findings.

Quantitative model component	Related qualitative themes	Explanatory contribution
Perceived teacher support and music learning motivation	Teacher support and encouragement; clear explanation; emotional care	Students reported that teachers' encouragement, praise, patience, and targeted guidance were important sources of support. This kind of support helped them build confidence, reduce anxiety, and learn music more easily.
Music learning motivation and music classroom engagement	Interest and enjoyable music-class experience; attractive classroom activities	Students reported that enjoyment, interest, performance opportunities, instrumental practice, video resources, and interactive activities in the classroom made them more willing to participate, concentrate, and engage in classroom interactions.
Indirect association among perceived teacher support, music learning motivation, and engagement	Content alignment with students' interests and abilities; participation opportunities	Students reported that they were more likely to develop learning interest and willingness to participate and engage in music classroom activities when teacher support was combined with activities and content that matched their interests and abilities.

Integration was gradually realized across the research design and interpretation stages. In the design stage, qualitative research was guided by quantitative results, focusing on how students understood teachers' support, motivation, and student engagement in music classrooms. At the level of interpretation, qualitative themes helped to explain statistical indirect associations: teacher support was experienced as emotional comfort and teaching help; these experiences were related to self-confidence, interest, and perceived ability; students then described engagement through active participation, concentration, performance, and interaction. Therefore, qualitative findings not only supported the quantitative results but also clarified the classroom process of understanding the observed association.

## 4 Discussion

This study investigated the associations among perceived teacher support, music learning motivation, and student engagement in Chinese junior high school music classrooms, with particular attention to the statistical role of learning motivation. The results indicate that perceived teacher support is positively associated with student engagement and music learning motivation, and that music learning motivation is also positively associated with student engagement. In addition, learning motivation shows a significant statistical indirect association in the relationship between perceived teacher support and student engagement. Overall, these findings suggest that student engagement is related to teacher support both directly and indirectly through motivational processes, while the cross-sectional design does not allow causal inferences or temporal ordering to be established.

First, the positive association between perceived teacher support and student engagement is consistent with a substantial body of prior research. When students perceive care, structure, and autonomy support from teachers, they are more likely to participate actively, maintain focus, and invest effort in learning activities (Skinner and Belmont, 1993; Klem and Connell, 2004). This finding is further supported by longitudinal studies, systematic reviews, and meta-analyses showing that teacher support is associated with students' engagement, motivation, and related learning outcomes (Hughes et al., 2008; Pedler et al., 2020; Yang et al., 2022; Tao et al., 2022; Vargas-Madriz et al., 2024; Martin et al., 2024; Zhang, 2024; Mammadov and Schroeder, 2023). In music classrooms, this association may be particularly meaningful because music learning often requires active participation, emotional expression, collaboration, and sustained attention. When students experience a supportive and encouraging instructional environment, they may be more inclined to participate in music classroom activities such as listening, singing, performing, discussing, and creating.

Second, perceived teacher support was positively associated with students' motivation for learning music. This finding aligns with both theoretical perspectives and empirical evidence indicating that teacher support is closely linked to the development of students' motivational beliefs and learning engagement over time (Held and Mori, 2024; Martin et al., 2024; Alrajeh and Shindel, 2020). In music education contexts, autonomy-supportive and emotionally responsive teaching practices have been associated with more positive learning experiences and outcomes (Bonneville-Roussy et al., 2020; Kiss et al., 2025). From a self-determination theory perspective, such supportive environments may facilitate the satisfaction of basic psychological needs, thereby fostering more adaptive forms of motivation (Ryan and Deci, 2002, 2017).

Third, music learning motivation was positively associated with student engagement in music classrooms. Students with higher levels of motivation tended to demonstrate greater participation, sustained attention, persistence, and emotional involvement in learning activities. This finding is consistent with theoretical perspectives suggesting that motivation reflects the reasons underlying students' initiation and persistence in learning behaviors, as well as the direction, intensity, and quality of their efforts, whereas engagement represents the observable manifestation of these motivational processes (Maehr and Meyer, 1997; Reeve et al., 2004). In this sense, motivation can be understood as a key psychological correlate of engagement. This result is also consistent with prior research in music education highlighting the central role of motivation in shaping students' persistence, participation, and learning experiences (Evans, 2015; Evans and Liu, 2019; Kiss et al., 2025).

The most notable finding of this study is that the indirect association between perceived teacher support and student engagement through learning motivation was statistically significant. This result suggests that the association between teacher support and engagement is partially linked to students' motivational experiences. However, given the cross-sectional design, this indirect-association pattern should be interpreted as a statistical association rather than evidence of causal mediation or temporal ordering. From the perspective of SDT, supportive learning environments may facilitate students' perceived autonomy, competence, and relatedness, which in turn are associated with more autonomous forms of motivation and higher levels of engagement (Ryan and Deci, 2002, 2017; Evans, 2015).

Similar indirect patterns have been observed in various educational contexts. Previous studies have shown that autonomy-supportive teaching is associated with identified regulation and behavioral engagement (Jang, 2008), and that the satisfaction of psychological needs has been examined as a mediator in the relationship between teacher support and classroom engagement (Zhou et al., 2022; Bureau et al., 2022). Related mediation models have also been reported in science education, technology-integrated learning environments, vocational education, and foreign language classrooms (Tas et al., 2019; An et al., 2022; Xu et al., 2023; Zhang and Hu, 2025). Furthermore, review studies suggest that teacher support may be related to engagement through multiple psychological pathways, including learning motivation, need satisfaction, and self-efficacy (Guo et al., 2023; Prananto et al., 2025). The present study extends this line of research by suggesting that a similar motivational pattern may be relevant within junior high school music classrooms.

Given the unique characteristics of music education, these findings should be interpreted within the specific context of music learning. Music education research suggests that learning motivation extends beyond the classroom and is related to how students engage with music in their daily lives, including their attentional focus and depth of musical experience (Harada and Takeishi, 2025). At the same time, focused and analytical listening is considered a fundamental activity in music learning, requiring sustained attention and cognitive engagement (Harada and Takeishi, 2025). This indicates that student engagement in music classrooms is strongly shaped by the nature of the learning activities themselves.

Moreover, music education theory highlights that teachers' choices of instructional materials, pedagogical strategies, and feedback practices shape students' perceptions of learning tasks, self-evaluations, and intrinsic motivation, and may be related to their continued participation in music learning (Asmus, 2021). At a more micro-level, teacher support is embedded in everyday classroom interactions. When students encounter difficulties in understanding or expression, teachers guide them to focus on key features, connect abstract concepts with concrete forms of expression, and provide scaffolding through questioning and explanation, thereby supporting sustained engagement (Ingulfsen et al., 2023). Recent qualitative research on Chinese university music classes has also reported that music-specific teacher scaffolding may involve demonstration, auditory modeling, guided listening, and timely feedback, and that such scaffolding was perceived by teachers as supporting students' autonomy, motivation, creativity, collaboration, and classroom supportiveness (Li and Wang, 2026).

In music classrooms, such support may take the form of providing opportunities for choice and expression, fostering collaboration and belonging, establishing clear learning goals, and offering appropriate assistance based on students' abilities. These practices are associated with higher levels of participation and sustained engagement (Schatt, 2024). The qualitative findings further suggest that the effectiveness of teacher support lies not only in students' perceived supportiveness, but also in their concrete classroom experiences, including encouragement, explanation, interaction, and alignment between teaching content and student needs. Through these experiences, teacher support is closely linked to students' confidence, interest, and willingness to participate. The interview results further suggest that students experienced teacher support in different forms. Emotional support was reflected in encouragement, patience, care, reassurance, and respectful communication, and was associated with students' self-confidence, reduced tension, and willingness to participate. Instructional support was reflected in clear explanations, targeted guidance, teacher demonstration, video-assisted teaching, and learning materials matched to students' ability levels. Students described this form of support as related to their understanding of classroom tasks and their participation in singing, performing, and other music classroom activities. In addition, performance opportunities, classroom interaction, and opportunities to express musical ideas reflected a participation-related aspect of teacher support in music classrooms. Because perceived teacher support was analyzed as an overall construct in the quantitative model, this study did not compare the separate statistical effects of different support types. Autonomy support was also not measured as a separate quantitative dimension; therefore, autonomy-related evidence was discussed cautiously through students' descriptions of participation opportunities, classroom interaction, and opportunities to express musical ideas. Overall, emotional support and instructional support appeared particularly salient in students' accounts, while participation-related support helped explain how teacher support was associated with music learning motivation and student engagement.

## 5 Conclusion

### 5.1 Summary of findings

This study examined the associations among perceived teacher support, music learning motivation, and student engagement in Chinese junior high school music classrooms. The results indicate a consistent statistical pattern in which higher perceived teacher support is associated with higher levels of music learning motivation and student engagement.

More specifically, students who reported higher levels of teacher support were more likely to demonstrate stronger engagement in music classroom activities. At the same time, teacher support was closely associated with students' motivation for music learning, which was also associated with higher levels of engagement. These findings suggest that student engagement in music classrooms was associated not only with instructional conditions but also with students' motivational processes in supportive learning environments.

The qualitative findings further elaborate this pattern by illustrating how students interpret teacher support in authentic classroom contexts. Support was consistently described in terms of encouragement, clarity of instruction, responsiveness, and respect for student expression. These experiences were perceived as facilitating task completion and were also associated with increased confidence, reduced anxiety, greater willingness to participate, sustained attention, effort, and emotional involvement in music learning.

The findings highlight the specific nature of music classrooms as contexts characterized by active participation, emotional expression, collaboration, and performance-based learning. In such settings, teacher support is not only related to task completion, but also associated with students' confidence to perform, interact, and express musical ideas publicly. This may explain why students identified encouragement, emotional care, clear instruction, opportunities for performance, and appropriately structured learning materials as meaningful forms of support.

The qualitative findings further suggested that students experienced teacher support mainly through emotional support, instructional support, and participation-related opportunities. Emotional and instructional support appeared particularly salient in students' accounts, while autonomy-related evidence was discussed cautiously because autonomy support was not measured as a separate dimension in the quantitative model.

Taken together, the results suggest that teacher support is embedded in students' learning processes not simply as an external condition, but as a classroom experience associated with how students perceive learning tasks, how they feel about participation, and how they maintain their own engagement in music classroom activities.

### 5.2 Theoretical implications

The findings of this study contribute to the literature by situating the relationship between teacher support and student engagement within the specific context of music education, thereby extending prior research that has largely focused on general academic subjects. By examining these relationships in music classrooms, the study provides context-sensitive evidence that highlights the role of subject-specific learning characteristics in shaping engagement processes.

In addition, the results are consistent with a process-oriented understanding of engagement by suggesting that learning motivation may be an important psychological variable associated with the relationship between external support and observable learning behavior. This perspective moves beyond a simple direct-association model and emphasizes the importance of internal motivational dynamics in understanding how instructional environments are related to student participation.

The integration of quantitative and qualitative findings further strengthens this contribution. While the quantitative results establish the structural relationships among variables, the qualitative data illuminate how these relationships are experienced and constructed by students in authentic classroom contexts. This combined perspective offers a more comprehensive account of engagement as a situated and meaning-making process rather than a purely measurable outcome.

This research contributes to the theoretical discussion of student engagement in music education. By examining teacher support, music learning motivation, and student engagement, this study extends SDT-related research to the general school music education context. The contribution is not limited to testing an existing model in a new context. As shown in the above examples, supportive teaching is often experienced in music classes through concrete musical activities, including performance opportunities, interaction opportunities, vocal and instrumental activities, video-supported learning, and teaching content that matches students' musical abilities and interests. This study also provides evidence from a non-Western educational context and investigates how teacher support, music learning motivation, and student engagement are connected with their actual experiences in the classroom. The results can offer comparative evidence for international research on motivation and engagement in music education.

Finally, the study contributes to the theoretical understanding of engagement formation in music education by showing that engagement is related not only to instructional design and activity structure but also to students' subjective interpretations of teacher behavior and the motivational meanings they attach to learning experiences.

### 5.3 Practical implications

The findings suggest that fostering student engagement in music classrooms requires a shift from a content-centered approach toward a more experience-oriented and student-responsive teaching perspective. While the development of musical knowledge and skills remains essential, the extent to which students are willing to participate, express themselves, and persist in learning appears to depend heavily on how they perceive the classroom environment and their relationship with the teacher.

Teacher support emerges as an important element in this process, not only in terms of instructional guidance but also in relation to students' emotional and motivational experiences. Practices such as providing clear explanations, offering constructive feedback, encouraging participation, and responding to students' individual needs appear to create conditions in which students feel more confident and willing to engage.

The qualitative findings highlight that students are particularly responsive to forms of support that are visible and personally meaningful, such as opportunities for expression, recognition of effort, and a sense of being understood. These experiences were associated with students' reports of a learning environment in which they felt more willing to take initiative, remain focused, and sustain their involvement in music activities.

From a broader instructional perspective, creating a classroom climate that supports autonomy, fosters positive relationships, and aligns learning activities with students' interests and abilities may be especially important in music education. Such conditions are not only associated with students' immediate engagement but may also be relevant to their motivation and willingness to continue participating in music learning.

### 5.4 Limitations and future directions

Several limitations should be acknowledged when interpreting the findings of this study. First, the use of a cross-sectional design limits the ability to establish causal relationships or temporal ordering among the variables. Although the proposed model is theoretically grounded and consistent with the data, it should be interpreted as a statistical indirect association. Longitudinal or experimental approaches may provide a more robust basis for examining the directionality and stability of these relationships over time. The alternative mediation models also showed some indirect effects under different arrangements of the variables. These results do not contradict the proposed model, but they show that cross-sectional data cannot determine the direction of causality.

Second, the reliance on self-reported data introduces the possibility of common method bias. Although the Harman single-factor test and the full collinearity VIF test results did not indicate serious common method bias, this concern cannot be fully ruled out. Future research may benefit from incorporating multiple data sources, such as classroom observations, teacher reports, or behavioral measures of engagement, in order to develop a more comprehensive understanding of student learning processes.

Third, the sampling structure should be considered when interpreting the research results. Students were drawn from four schools, and the data may therefore involve school-level or class-level clustering, which could influence the estimation of standard errors. The existing dataset included school identifiers. An exploratory school-level inspection was conducted. The results showed that the intraclass correlation coefficients (ICCs) for perceived teacher support, music learning motivation, and student engagement in music classrooms were very low. However, only school identifiers were available in this analysis. Class- and teacher-level information was not recorded. Therefore, it was not possible to directly model potential dependence at the class or teacher level. Future research should collect class and teacher identifiers and use multilevel analysis or cluster-adjusted analyses when appropriate.

The study is situated within a specific cultural and educational context, which may limit the generalizability of the findings. Further research across different regions, age groups, and instructional settings would help to examine the extent to which the observed relationships hold in other contexts.

In addition, although learning motivation was identified as an important psychological variable in the statistical indirect-association model, other psychological and contextual factors may also play important roles. Future studies could explore additional pathways, including basic psychological need satisfaction, academic emotions, self-efficacy, and peer interaction, as well as examine potential differences across types of motivation.

Expanding the qualitative component of the research may also provide deeper insights into how students interpret and respond to classroom experiences. Including a more diverse range of participants and extending the scope of qualitative inquiry could further enrich the understanding of engagement processes in music education.

## Data Availability

The raw data supporting the conclusions of this article will be made available by the authors, without undue reservation.
